# Chemically Cross-Linked Cellulose Nanocrystal Aerogels for Effective Removal of Cation Dye

**DOI:** 10.3389/fchem.2020.00570

**Published:** 2020-07-07

**Authors:** Luna Liang, Shuyang Zhang, Gabriel A. Goenaga, Xianzhi Meng, Thomas A. Zawodzinski, Arthur J. Ragauskas

**Affiliations:** ^1^Department of Chemical and Biomolecular Engineering, University of Tennessee, Knoxville, Knoxville, TN, United States; ^2^Chemical Sciences Division, Oak Ridge National Laboratory, Oak Ridge, TN, United States; ^3^Department of Forestry, Wildlife, and Fisheries, Center for Renewable Carbon, University of Tennessee Institute of Agriculture, Knoxville, TN, United States; ^4^Joint Institute for Biological Science, Biosciences Division, Oak Ridge National Laboratory, Oak Ridge, TN, United States

**Keywords:** cellulose nanocrystals, aerogel, chemical cross-linking, adsorption, cation dye

## Abstract

In this study, porous aerogels were prepared by directional freeze-drying via cross-linking cellulose nanocrystals (CNCs) with poly(methyl vinyl ether-co-maleic acid) (PMVEMA) and poly(ethylene glycol) (PEG). The thermal properties and physical adsorption performance toward cation methylene blue dye of the obtained CNC aerogels were investigated. The maximum degradation temperature was increased from 324°C of CNCs to 355°C of cross-linked CNC aerogels. The dye adsorption isotherm results showed that the maximum methylene blue adsorption capacity of CNC aerogels was 116.2 mg g^−1^, according to the Langmuir model, which was mainly due to the electrostatic attractions between negatively charged carboxyl groups or sulfonate groups on the CNC aerogles and cation MB molecules. The reusability test showed that the CNC aerogels contained the same dye adsorption performance in five adsorption/desorption cycles. Overall, this study described an ideal alternative for water purification with high dye adsorption capacity and enhanced physical performance.

## Introduction

Currently, more than 9,000 types of dyes have been incorporated in a variety of industries, such as textile, leather, printing, and cosmetics (Garg et al., 2003; Tan et al., 2015). Synthetic dyes have complex structures and low biodegradability, which are difficult to be eliminated from contaminated water streams and may have harmful properties, including teratogenic, carcinogenic, and mutagenic effects on human health (Yang et al., 2016). They may also decrease the sunlight penetration into the water and interfere the photosynthetic activity of aquatic life (Safa and Bhatti, 2011). Therefore, it is an important and challenging task to remove dyes from industrial process streams before they are discharged into the environment. Several approaches such as biodegradation (Ghoreishi and Haghighi, 2003), advanced oxidation (Mandal et al., 2010), photo-catalysis (Palácio et al., 2012), coagulation/flocculation (Nkurunziza et al., 2009; Wu et al., 2011), membrane separation (Avlonitis et al., 2008), and adsorption (Kamal et al., 2016) are used in the removal of dyes from wastewater. However, many disadvantages are coupled with these dye removal techniques. For example, the biodegradation is strongly dependent on the operation conditions and the oxidation requires highly efficient oxidative agents (Kamal et al., 2016). Among these approaches, the decolorization of wastewaters by adsorption is considered as a simple and economical process for removing dyes from wastewater using inexpensive and efficient solid supports (Jin et al., 2015). Various adsorbents are used in water purification, such as activated carbon, agricultural solid wastes, industrial by-products, clay minerals, biomass, and polymeric resins (Salleh et al., 2011; Wang et al., 2015; Gamoudi and Srasra, 2018; Hasanzadeh et al., 2020). Based on the high porosity and large surface area, aerogels have been reported to be promising alternatives in water purification (Bi et al., 2013; Wu et al., 2013).

Aerogels are flexible and highly porous materials usually derived by a solvent exchange, freeze-drying, or supercritical drying process (Yang and Cranston, 2014; Zu et al., 2016; De France et al., 2017). Aerogels were first synthesized in the 1930s with the pioneering work of Kistler using either inorganic materials (i.e., silica, alumina, or tungstic oxide) or biopolymers (i.e., cellulose, nitrocellulose, or gelatin) (Kistler, 1932). However, aerogels based on silica or other inorganic nanoparticles often show fragility which limit their applications. Nowadays, cellulose nanocrystals (CNCs) have been reported to be an economical and eco-friendly alternative in the aerogel component with their lightweight, strong mechanical strength, and safe handling compared to synthetic nanoparticles. Due to the high specific surface area and broad possibility of surface modification, CNCs also show the promising potential application of biobased adsorbent in environmental remediation such as organic pollutants removal, oil removal and dye removal from waste effluents (Mahfoudhi and Boufi, 2017; Wang et al., 2018). CNCs, also called cellulose whiskers or nanowhiskers, are well-defined crystalline rigid rodlike particles with low density (1.59 g cm^−3^), liquid-crystalline properties, biodegradability, and biocompatibility (De France et al., 2017; Xu et al., 2018). The dimension of CNCs varies depends on the type of cellulosic source and treatment process but usually in the range of 5–15 nm in width and 100–1,000 nm in length (Moon et al., 2011; Jordan et al., 2019; Xu et al., 2019).

Therefore, the challenge of creating high-performance CNC aerogels by simple routes has become an active field of study. A chemical cross-linking method could be employed to form effective covalent bonds based on the numerous active hydroxyl groups on the surface of CNCs to advance the structure stability and mechanical performance of CNC-based aerogels (Zhu et al., 2019; Liang et al., 2020). Poly(methyl vinyl ether-co-maleic acid)/poly(ethylene glycol) (PMVEMA/PEG) hydrogels have been investigated to have a high swelling capability and have been reported to be used as cross-linking agents to make nanocellulose composites (Goetz et al., 2009; Raj Singh et al., 2010). Herein, we report a simple route to form chemically cross-linked PMVEMA/PEG/CNC aerogels via a directional-freezing technique and freeze-drying process followed by thermal treatment. The achieved CNC aerogels showed enhanced thermal properties. The negatively charged carboxyl groups from PMVEMA and the sulfonate groups from CNCs made it possible for the CNC aerogels to become efficient adsorbents for cation dye removal. Thus, the methylene blue (MB) dye removal performance of the obtained CNC aerogels as renewable adsorbents was evaluated and compared to other reported adsorbents from literature.

## Materials and Methods

### Materials

Poly(methyl vinyl ether-*alt*-maleic acid) (PMVEMA) with M_w_ of ~216,000 was purchased from Sigma-Aldrich (St. Louis, MO, USA). Polyethylene glycol 4000 (PEG) was obtained from Alfa Aesar (Haverhill, MA, USA). CNCs extracted from wood pulp with 10.3 wt% solid content were provided by The University of Maine (Orono, ME, USA) and used without further purification. Methylene blue (MB) was of chemical grade and purchased from Fisher Scientific (Fair Lawn, NJ, USA). All other chemicals were obtained from Fisher Scientific and used without further treatment.

### Preparation of Cross-Linked Nanocomposite Aerogels

The preparing procedure of cross-linked PMVEMA/PEG/CNC aerogels has already been described in our previous paper (Liang et al., 2019). The mass ratio on PMVEMA: PEG was maintained as 6.7:1. The composition of CNC aerogels is presented in Table 1. After directional freeze-drying, the CNC aerogels were thermally treated in a Lindberg/Blue hinged tube furnace (Thermo Scientific, Waltham, Ma, USA) at 250°C for 1 h under a nitrogen flow. After thermal treatment, the CNC samples were removed from the furnace and cool down to room temperature for further use.

**Table 1:** Composition of CNC aerogels.

**Sample coding**	**CNC, wt%**	**PMVEMA/PEG, wt%**
25CNC	25.00	75.00
50CNC	50.00	50.00
75CNC	75.00	25.00
100CNC	100.00	0.00

### Fourier Transform Infrared Spectroscopy (FT-IR)

Fourier transform infrared spectroscopy (FT-IR) were acquired using the PerkinElmer Spectrum 100 FT-IR Spectrometer (PerkinElmer, Waltham, MA, USA) at a resolution of 4 cm^−1^ with a range of 4,000–600 cm^−1^ at the accumulation of 32 scans.

### Surface Area and Pore Size Analysis

A Quantachrome Autosorb iQ and ASiQwin Instrument (Quantachrome Corporation, Boynton Beach, FL, USA) was used to evaluate the surface area and the pore size distribution of the materials. In brief, a sample (0.20–0.30 g) was placed into the BET cells for analysis. Samples were degassed at 150°C for 5 h, thus removing any moisture that could alter the weight of the samples. Experiments were performed by N_2_ adsorption/desorption technique at −196°C. Specific surface areas were calculated using the 11 points multi-point Brunauer-Emmett-Teller (BET) gas adsorption method, and pore size distribution was calculated using the Barrett-Joyner-Halenda (BJH) analysis during the desorption isotherm (Hayati-Ashtiani, 2011).

### Porosity

The porosity of samples was determined by the ethanol displacement method (Catanzano et al., 2018). The volume of the samples (*V*_*s*_) was calculated by measuring diameter and height using a digital caliper. The dry samples were first stored in a 90°C oven for 2 h to remove trace moisture and then weighted (*w*_0_). Then the samples were immersed in anhydrous ethanol for 24 h. The samples were taken out and gently blotted the sample surface with filter paper to remove excess ethanol on the surface. The samples were reweighted (*w*_1_) immediately. The porosity was then calculated according to the following Equation (1),

(1) $$\begin{array}{r} \text{Porosity }\left(\%\right)=\;\frac{w_{1}-w_{0}}{\rho_{e}V_{s}}*100\% \end{array}$$

where ρ_*e*_ is the density of anhydrous ethanol (0.79 g/cm^3^). The average value of duplicates for each sample was taken.

### Thermal Gravimetric Analysis (TGA)

Around 8.00–10.00 mg CNCs, CNC aerogels, PMVEMA/PEG mixture were evaluated using a TGA Q50 Thermo-Gravimetric Analyzer (TA Instruments, New Castle, DE, USA). Thermal gravimetric analysis (TGA) tests were initiated at room temperature to 800°C with a heating rate of 20°C min^−1^ under nitrogen atmosphere. The change in weight-related to temperature increase was recorded.

### Adsorption of Cation Dyes

The 25, 50, and 75CNC aerogels were immersed in 20.00 mL of methyl blue (MB) aqueous solutions with various concentrations (50–1,200 mg L^−1^) and stirred on an incubator at 150 rpm and at RT for 24 h. The aerogels were then removed from the solution, and the residual concentration of dye was determined by UV–vis Spectroscopy (Shimadzu Corporation, Kyoto, Kyoto, Japan) at 660 nm. The average values of three replicates of each sample were reported. A standard calibration curve was derived from a series of dye solutions with known dye content (Vilela et al., 2019). The linear calibration curve for MB was obtained in the range of 0.25–7.00 mg L^−1^ by *y* = 0.1891*x*+0.0584 (*R*^2^ = 0.992). The adsorbed amount of dyes was calculated according to the following Equation (2),

(2) $$\begin{array}{r} q_{t}=\frac{\left(C_{0}-C_{t}\right)V}{m} \end{array}$$

where q_t_ is the amount of dye adsorbed after time t, C_0_ and C_t_ are initial concentration and concentration of the adsorbate after time t, respectively, mg L^−1^; V is the volume of the solution, L, and m is the weight of the CNC aerogels used, g (He et al., 2013; Jin et al., 2015). The percentage of dye removal was calculated using the following Equation (3),

(3) $$\begin{array}{r} \%\text{Removal}=\frac{\left(C_{0}-C_{t}\right)}{C_{0}}*100 \end{array}$$

### Reusability of Adsorbent

The reusability of 25, 50, and 75CNC aerogels was investigated with modification to a literature procedure (Yang et al., 2016). Approximately, 0.10 g CNC aerogel was placed in a 10.00 mL MB solution (50.00 mg L^−1^) and stirred at 150 rpm for 2 h at RT. The concentration of MB was measured by UV-vis. The dye adsorbed on CNC aerogels was desorbed by immersing the aerogel in 20.00 mL 0.10 M HCl and agitating at 150 rpm with an incubator for 30 min, followed by washing with 20.00 mL 0.10 M NaOH, rinsing with water and finally rinsing with 10 mL ethanol and drying under air. The MB removal ability was calculated using Equation (2). The adsorption-desorption procedure was repeated five times and the average values of three replicates of each sample were reported.

## Results and Discussion

### Characteristics of CNC Aerogels

**Figure 1:**
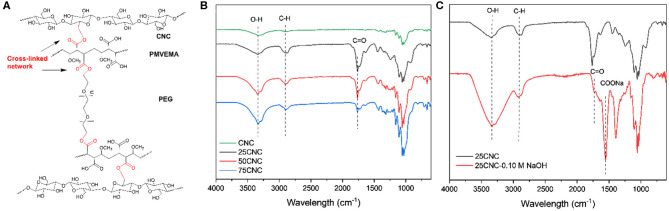
**(A)** Scheme of the possible structure of thermal treated cross-linked CNC aerogels. **(B)** FT-IR spectra of CNC aerogels. **(C)** FT-IR spectra of NaOH treated and un-treated 25CNC aerogels.

The cross-linking mechanism for thermally treated CNC aerogels is shown in Figure 1A. The FT-IR spectra (Figure 1B) of all the samples exhibited a broad peak centered at 3,300 cm^−1^ and a strong peak at 2,900 cm^−1^, which were assigned to the O-H and C-H stretching vibrations, respectively (Herrera et al., 2017). Cross-linked networks in CNC aerogels can be formed by the esterification reaction between hydroxyl group on CNC surface, terminal hydroxyl groups of PEG, and carboxylic acid group on the PMVEMA during the thermal treatment. All cross-linked CNC aerogels showed a new peak at 1,768 cm^−1^, which is assigned to the combination of both the ester carbonyl stretch from the ester-linkages cross-linked CNC aerogels and from the unreacted carboxylic acid functional groups from the PMVEMA (Goetz et al., 2009). In order to eliminate the influence of ester linkages and unreacted carboxyl groups, the aerogels were treated with 0.10 M NaOH. Figure 1C shows the FT-IR spectra of NaOH treated and un-treated 25CNC aerogels. A new peak at 1,555 cm^−1^ appeared in NaOH treated 25CNC spectrum, which is assigned to the carboxylate carbonyl resulting from the dilute base treatment. Thus, the observed ester peak at 1,740 cm^−1^ can then be attributed to the formation of the ester linkages between PMVEMA and CNC or PEG. Comparing to our previous paper (Liang et al., 2019), the same cross-linking mechanism was observed of CNC aerogels when thermal treated at 90 and 250 (Figure S1). The slight difference was that the peak of ester carbonyl stretch and carboxylic acid groups was observed at 1,768 cm^−1^ when treated at 250°C and when treated at 90°C a peak was observed at 1,720 cm^−1^. This may be attribute to the dehydration of the diacids on the PMVEMA at 166.16°C (Aguirre et al., 2011), which was discussed in the thermal analysis.

The density, BET specific surface area (S_BET_), BJH pore volume (V_BJH_) and porosity of aerogels were also examined in this study. The density of CNC aerogels was in the range of 0.08–0.11 g cm^−3^ and slightly increased with increasing CNC content. The result may be due to the weight loss of the PMVEMA component during the thermal treatment, which was proved by the thermal analysis results. The weight loss also showed that the density of the aerogels could be controlled by selecting CNC concentration in the suspension in the preparation process. Other than that, the surface area and pore volume were investigated by the adsorption/desorption of nitrogen through BET analysis (Table 2, Figure S2). The BET results indicated that cross-linked aerogels had a larger surface area. For example, the S_BET_ values were 8.23 m^2^ g^−1^ for 100CNC and around 17.00 m^2^ g^−1^ for cross-linked aerogels. Also, the cross-linked CNC aerogels had shown larger pore volumes. The larger surface area and pore volume could provide more adsorption sites and hence are expected to enhance dye removal ability. In our previous study, the structure of CNC aerogels made by directional freezing technique have been investigated and the results indicated that those aerogels had organized lamellar structure (Liang et al., 2019). The mesoporosity of these CNC aerogels is also confirmed by the pore size distribution analysis (Figure S2) (Sing, 1985). The porosity was calculated via the ethanol displacement method, in which ethanol is a solvent that able to penetrate material pores easily without changing the geometrical volume of the aerogel. The result showed that CNC aerogels contained a consistent high porosity of around 86%.

**Table 2:** Physical parameters of CNC aerogels.

	**Density (g cm^**−3**^)**	**BET specific surface area (m^**2**^ g^**−1**^)**	**BJH pore volume (m^**3**^ g^**−1**^)**	**Porosity (%)**
25CNC	0.0877 ± 0.0003	16.86	0.045	86.83 ± 4.07
50CNC	0.0912 ± 0.0004	16.29	0.050	86.60 ± 0.73
75CNC	0.1005 ± 0.0014	17.00	0.024	86.12 ± 1.43
100CNC	0.1105 ± 0.0007	8.23	0.020	85.96 ± 1.46

### TG and DTG Analysis

**Figure 2:**
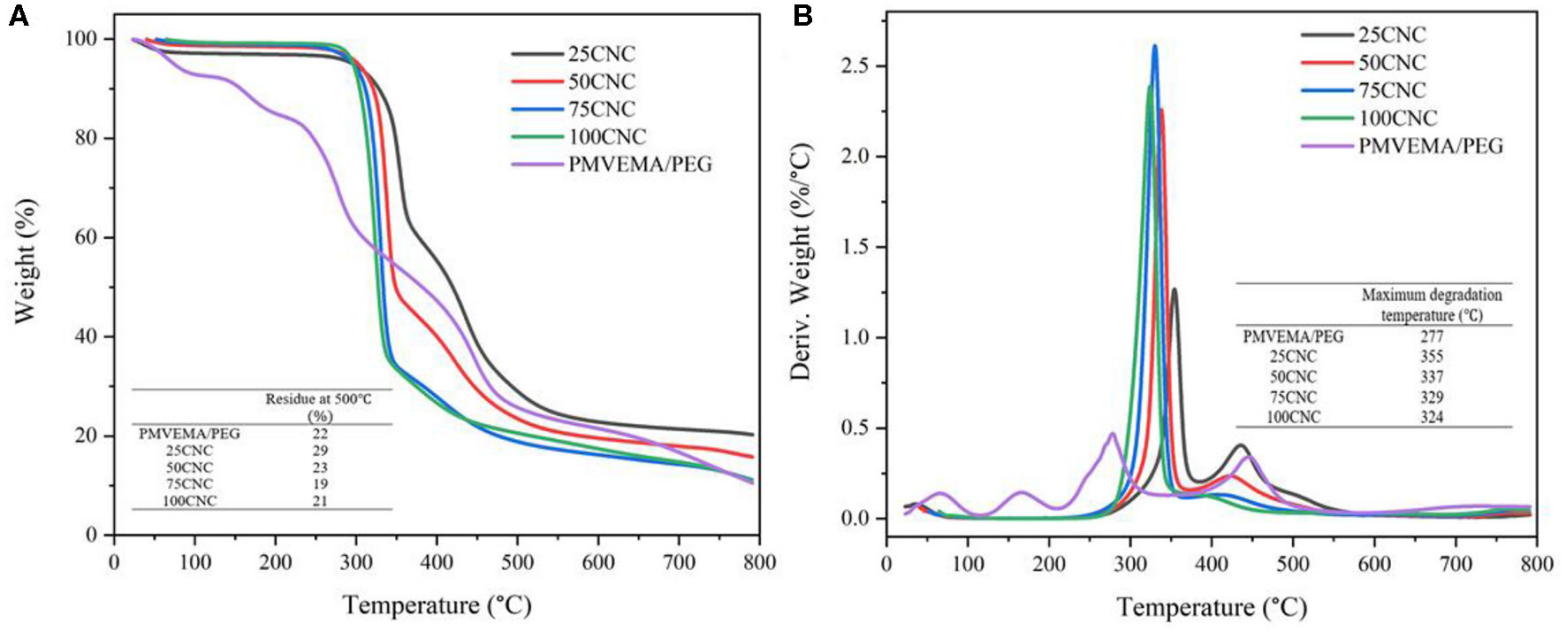
TGA **(A)** and DTG **(B)** curves for cross-linked CNC aerogels and PMVEMA/PEG mixture.

Thermogravimetric (TG) and derivative TG (DTG) curves for cross-linked CNC aerogels are summarized in Figure 2. This procedure was performed to evaluate how the cross-linking process affected the thermal stability of the samples. The DTG curves of PMVEMA/PEG showed four distinct peaks. The first peak at 66°C is associated with the loss of water content. The second peak at 166°C is associated with the dehydration of the diacids of the PMVEMA. The third peak at 277°C is corresponding to the combined decomposition of PMVEMA and PEG, and the final peak at 445°C is attributed to the polymer chain degradation (Nair et al., 2014). For the cross-linked CNC aerogels and pure CNCs, a significant weight loss was observed in the range of 270–460°C, corresponding to the depolymerization of polymer chain scission and cross-link breakage (Abraham et al., 2013). The DTG results showed that the weight loss of CNC aerogels after thermal treatment which was attributed to the dehydration of the diacids of the PMVEMA. The primary degradation peak centered at 324°C for 100CNC, 329°C for 75CNC, 337°C for 50CNC, and 355°C for 25CNC, which indicated a better thermal stability of the cross-linked CNC aerogels compared to 100CNC aerogels. The formation of cross-linkages limited the mobility of the polymer chains, which had a positive influence on the thermal stability (Liang et al., 2020). Moreover, the maximum decomposition rate (2.62% °C^−1^ of 75CNC, 2.25% °C^−1^ of 50CNC, and 1.27% °C^−1^ of 25CNC) was decreased with increasing content of the cross-linking agent, which indicates that the 25CNC aerogel is more thermally stable compare to 50CNC or 75CNC aerogel due to the higher cross-linking density. Moreover, the char residues at 500°C increased from 19 wt% (75CNC) to 29 wt% (25CNC) with increasing cross-linking agent content. The increase in char residues could be ascribed to the formation of cross-linkages and lower decomposition rates, which is consistent with previous results (Nair et al., 2014; Jiang and Hsieh, 2017). In summary, the thermal stability of CNC aerogels was improved by the formation of cross-linkages.

### Adsorption Isotherms of CNC Aerogels With Cation Dyes

**Figure 3:**
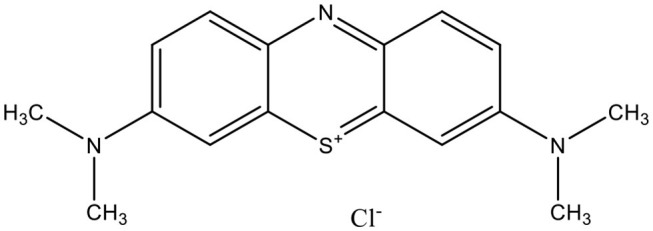
Structure of MB.

**Figure 4:**
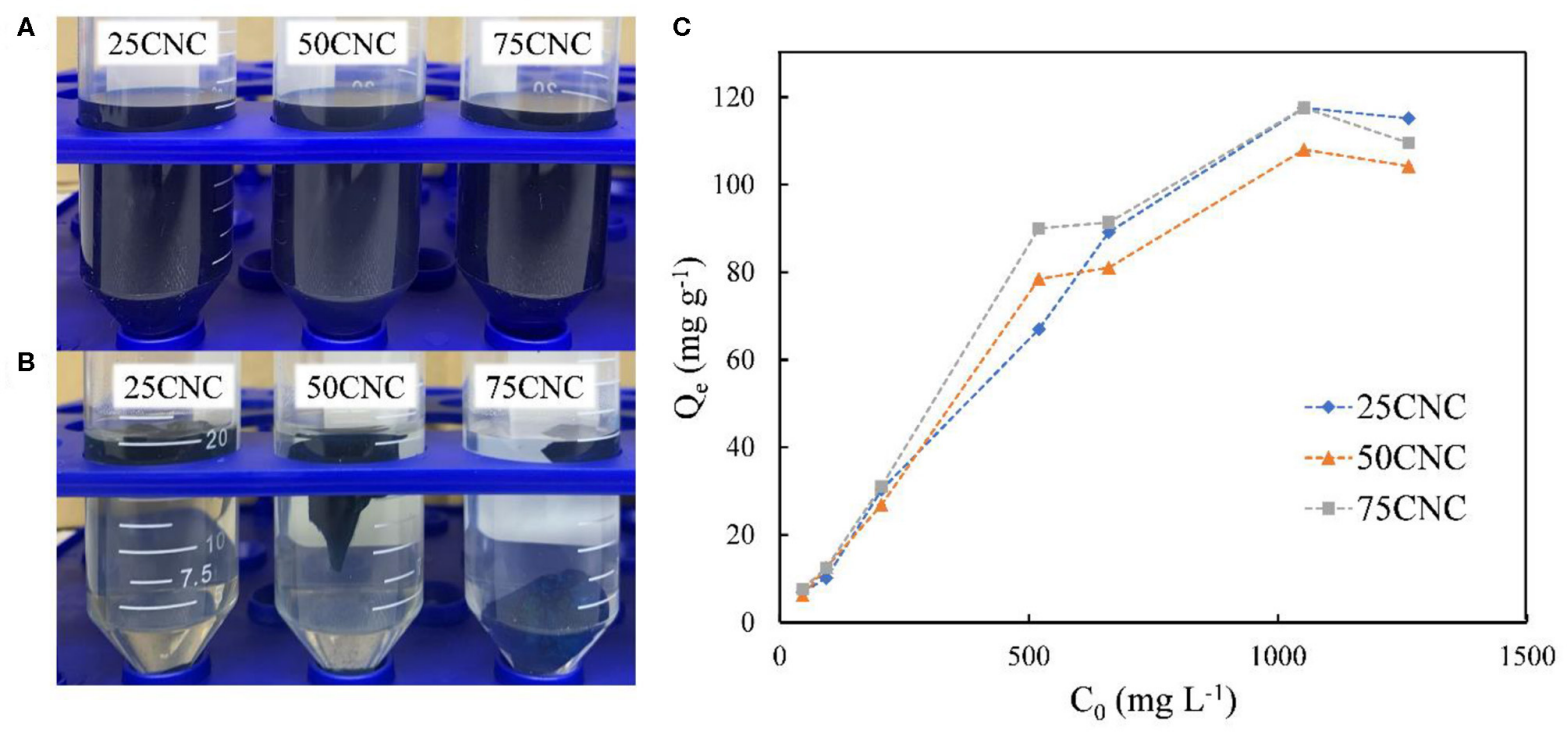
100 mg L^−1^ MB dye solution before **(A)** and after **(B)** CNC aerogels adsorption. The dye removal ability of CNC aerogels from 50 to 1,200 mg L^−1^ MB solution **(C)**.

To demonstrate the potential dye adsorption property of the cross-linked CNC aerogels, a cationic dye MB (Figure 3) was used in the dye adsorption experiment. MB is a heterocyclic cationic aromatic compound that is used either as a dye or a drug with, for example, antimalarial, antidepressant, and anxiolytic activity, which is potentially toxic toward humans and environment (Vilela et al., 2019). An example of MB removal efficiency is shown in Figures 4A,B. The initial MB concentration provides a significant driving force to overcome mass transfer resistance of the molecules between the aqueous and the solid phases (Kumari et al., 2016). Figure 4C shows the relationship between the initial MB concentration and MB adsorption at equilibrium by CNC aerogels from aqueous solution. After 24 h adsorption, the obtained results showed that Q_e_ values increased from 6.92 to 115.15 mg g^−1^ for 25CNC aerogels with an increase in MB concentration from 50.00 to 1200.00 mg L^−1^. A similar trend also observed for 50CNC and 75CNC aerogels. These findings can be attributed to the high ratio of absorbent sites available to the dye molecules at lower MB concentrations and the saturation of adsorption sites on CNC aerogels at higher MB concentrations (Kumari et al., 2016; Melo et al., 2018).

**Figure 5:**
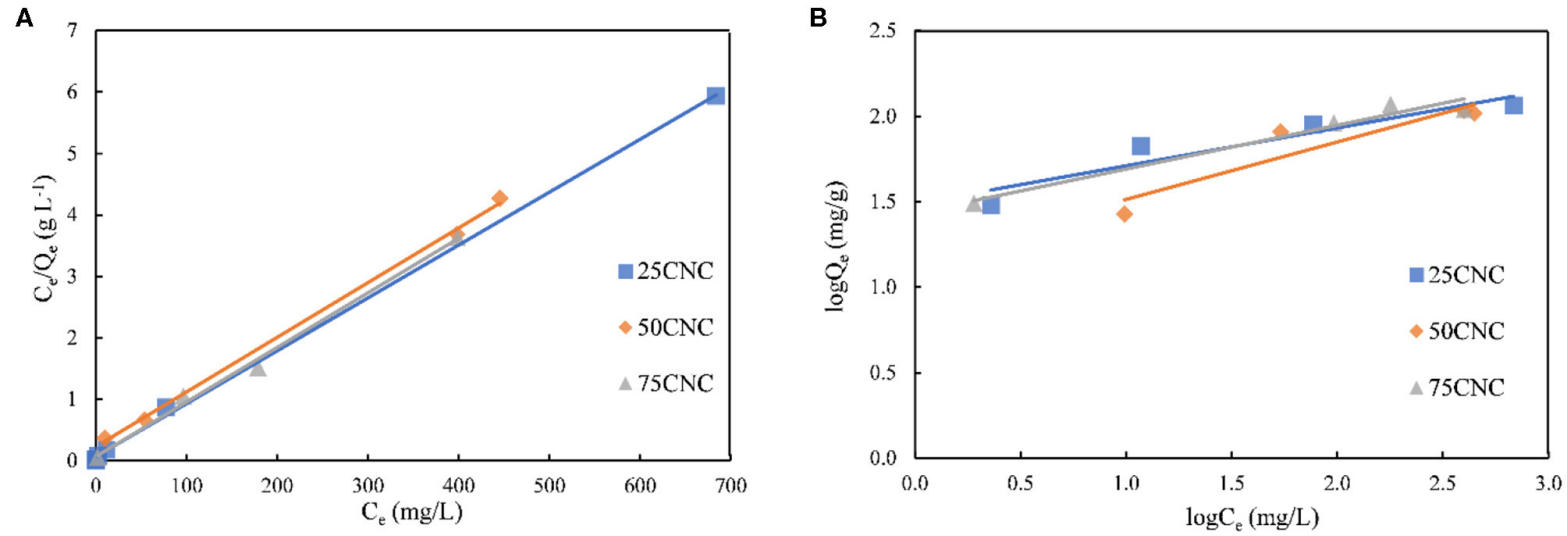
The linear fit of Langmuir model **(A)**. The linear fit of Freundlich model **(B)**.

The adsorption isotherm is an important tool to describe how adsorbate molecules interact with an adsorbent surface. The Langmuir and Freundlich model were used to investigate the relationship between the aerogel and MB molecules. The Langmuir isotherm model is based on monolayer adsorption onto a surface with a finite number of adsorption sites of uniform adsorption energies (Jin et al., 2015). It can be expressed as Equation (4):

(4) $$\begin{array}{r} \frac{C_{e}}{Q_{e}}=\frac{1}{Q_{m}K_{L}}+\frac{C_{e}}{Q_{m}} \end{array}$$

where *Q*_*e*_ is the solid phase adsorbate concentration at equilibrium (mg g^−1^), *Q*_*m*_ is the maximum adsorption capacity corresponding to complete monolayer coverage on the surface (mg g^−1^), *C*_*e*_ is the concentration of adsorbate at equilibrium (mg L^−1^), and *K*_*L*_ is the Langmuir constant (L mg^−1^). The linear Langmuir plots for CNC aerogels adsorption of MB are shown in Figure 5A. *Q*_*m*_ and *K*_*L*_ were calculated from the slope and intercept of the linear fitting curve, respectively.

The Freundlich equation is an empirical equation employed to describe heterogeneous systems, in which it is characterized by the heterogeneity factor 1/*n* (Hameed et al., 2007), the Freundlich expression can be obtained by taking logarithms of Equation (5):

(5) $$\begin{array}{r} lnQ_{e}=lnK_{F}+\frac{1}{n}lnC_{e} \end{array}$$

where *K*_*F*_ is the Freundlich constant (mg g^−1^) (L mg^−1^)^1/n^ and 1*/n* is the heterogeneity factor. The linear Freundlich adsorption isotherm fit is shown in Figure 5B.

The linear analysis of Langmuir and Freundlich adsorption isothermal models indicated that the observed dye adsorption data for cross-linked CNC aerogels could be better described by the Langmuir isotherm model as confirmed by a higher coefficient *R*^2^ (Table 3). The Langmuir fitting results suggested that the adsorption process between CNC aerogels and MB dye could be characterized as a monolayer type of adsorption. The theoretical maximum adsorption capacity of CNC aerogels calculated by Langmuir model was 116.20, 112.40, and 112.60 mg g^−1^ for 25CNC, 50CNC, and 75CNC, respectively. The negatively charged carboxyl groups from PMVEMA and sulfonate groups from CNCs on the aerogel provided available sites for binding cationic MB dye molecules to the aerogel through electrostatic attraction (Yang et al., 2016). A direct comparison of the maximum adsorption capacities of MB dye by various adsorbents is presented in Table 4. Batmaz et al. reported the maximum MB adsorption of carboxylated CNC is 769.00 mg g^−1^, and the higher removal capacity is most likely associated with the introduced negative charges onto CNCs, which increased the adsorption sites (Batmaz et al., 2014). Still, the dye removal values of the CNC aerogels prepared in the present study are comparable, for instance, with Fe_3_O_4_/activated montmorillonite or magnetic cellulose/graphene oxide, listed in Table 4. Furthermore, the achieved CNC aerogels are water-stable, which indicated that it could be readily removed and recycled after water purification. Hence, the study of dye adsorption suggested that the cross-linked CNC aerogels are highly suitable for the removal of cation dyes from the aqueous solutions.

**Table 3:** Langmuir and Freundlich isothermal adsorption constant, equation, and regression coefficient of the linearized plot for the adsorption of MB.

	**Langmuir model**				**Freundlich model**			
	**Q_**m**_ (mg g^**−1**^)**	***K***	**Equation**	***R^**2**^***	***K_***F***_***	***n***	**Equation**	***R*^**2**^**
25CNC	116.20	0.125	*y* = 0.0086*x*+0.0686	0.999	20.89	0.309	*y* = 0.2214*x*+1.489	0.881
50CNC	112.40	0.039	*y* = 0.0089*x*+0.2299	0.998	15.04	0.336	*y* = 0.336*x*+1.1774	0.869
75CNC	112.60	0.130	*y* = 0.0089*x*+0.0684	0.994	27.20	0.257	*y* = 0.2566*x*+1.4345	0.965

**Table 4:** The maximum MB adsorption capacity of different adsorbents.

**Absorbent**	**Maximum capacity (mg g^**−1**^)**	**References**
Natural raw (Algerian) kaolin	52.76	Mouni et al., 2018
Activated carbons from Sunflower oil cake	16.43	Karagöz et al., 2008
Graphene	153.85	Liu et al., 2012
Fe_3_O_4_/activated montmorillonite	106.38	Chang et al., 2016
Cellulose nanowhiskers/polyurethane foam	110.50	Kumari et al., 2016
Magnetic cellulose/graphene oxide	70.03	Shi et al., 2014
Carboxylated CNC	769.00	Batmaz et al., 2014
PMVEMA/PEG/CNC	116.20	This work

### Reusability Test

**Figure 6:**
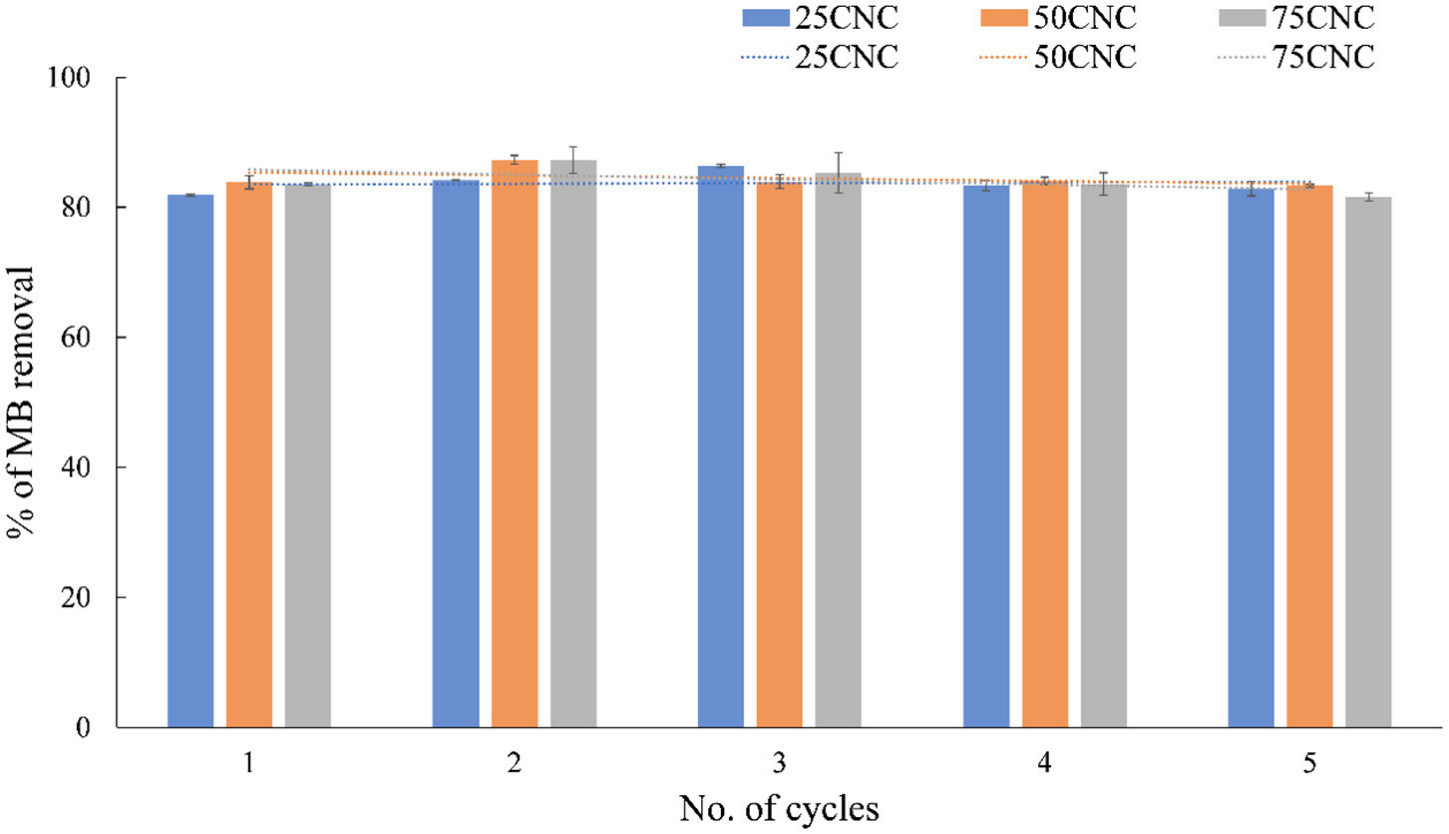
Reusability tests of CNC aerogels for the adsorption efficiency toward MB dyes.

The stability and reusability are significant for an ideal adsorbent in practical application, which will significantly increase the efficiency and reduce the cost (Fang et al., 2018). The reusability test was studied by evaluating the MB removal efficiency (%) for 5 consecutive cycles in Figure 6. The result indicated that the adsorbent conserved an excellent activity in five cycles of reuse. A slight decrease trend in MB adsorption performance may be due to the part of preabsorbed MB particles, which are trapped in the aerogel structure, reducing the total available negatively charged carboxyl groups for subsequent adsorption cycles (Yang et al., 2016). Moreover, the CNC aerogels contained their original shape after MB adsorption, which can be easily removed and recycle.

## Conclusions

In summary, porous CNC aerogels were successfully fabricated by cross-linking with PMVEMA/PEG matrix. Based on the advantages of the highly porous structure, improved thermal stability and dye adsorption ability, CNC aerogels were ideal alternatives to be used as absorbents to remove cation dyes from aqueous solution. Furthermore, 25CNC aerogels show the best thermal stability and adsorption capacity toward MB dye among the three aerogels made with different concentrations of CNCs. In addition, the adsorption of CNC aerogels still maintained almost the same performance in five adsorption/desorption cycles.

## Data Availability Statement

The original contributions presented in the study are included in the article/Supplementary Materials, further inquiries can be directed to the corresponding author/s.

## Author Contributions

SZ tested the dye adsorption reusability. GG measured the surface area and pore volume. XM advised the dye adsorption study. TZ and AR supervised this work. LL performed thermal and dye adsorption experiments and analysis. The manuscript was written by LL. The manuscript was revised by all the authors.

## Conflict of Interest

The authors declare that the research was conducted in the absence of any commercial or financial relationships that could be construed as a potential conflict of interest.
