# Supplementary material for: Chemically Cross-Linked Cellulose Nanocrystal Aerogels for Effective Removal of Cation Dye
Source: Front Chem. 2020 Jul 7;8:570. doi: 10.3389/fchem.2020.00570 (PMC7359072; doi:10.3389/fchem.2020.00570)
Supplement: Supplementary file 1 [file Table_1.DOCX]

**Chemically cross-linked cellulose nanocrystal aerogels for effective removal of cation dye**

Luna Liang^1^, Shuyang Zhang^1^, Gabriel A. Goenaga^1^, Xianzhi Meng^1^, Thomas A. Zawodzinski^1,2^, and Arthur J. Ragauskas^1,3,4^

^1^Department of Chemical & Biomolecular Engineering, University of Tennessee Knoxville, Knoxville, TN 37996 (USA)

^2^Chemical Sciences Division, Oak Ridge National Laboratory. Oak Ridge, TN 37831 (USA)

^3^Department of Forestry, Wildlife, and Fisheries, Center for Renewable Carbon, University of Tennessee Institute of Agriculture, Knoxville, TN 37996 (USA)

^4^UTK-ORNL Joint Institute for Biological Science, Biosciences Division, Oak Ridge National Laboratory, Oak Ridge, TN 37831 (USA)

Correspondence:

Dr. Arthur J. Ragauskas

aragausk@utk.edu


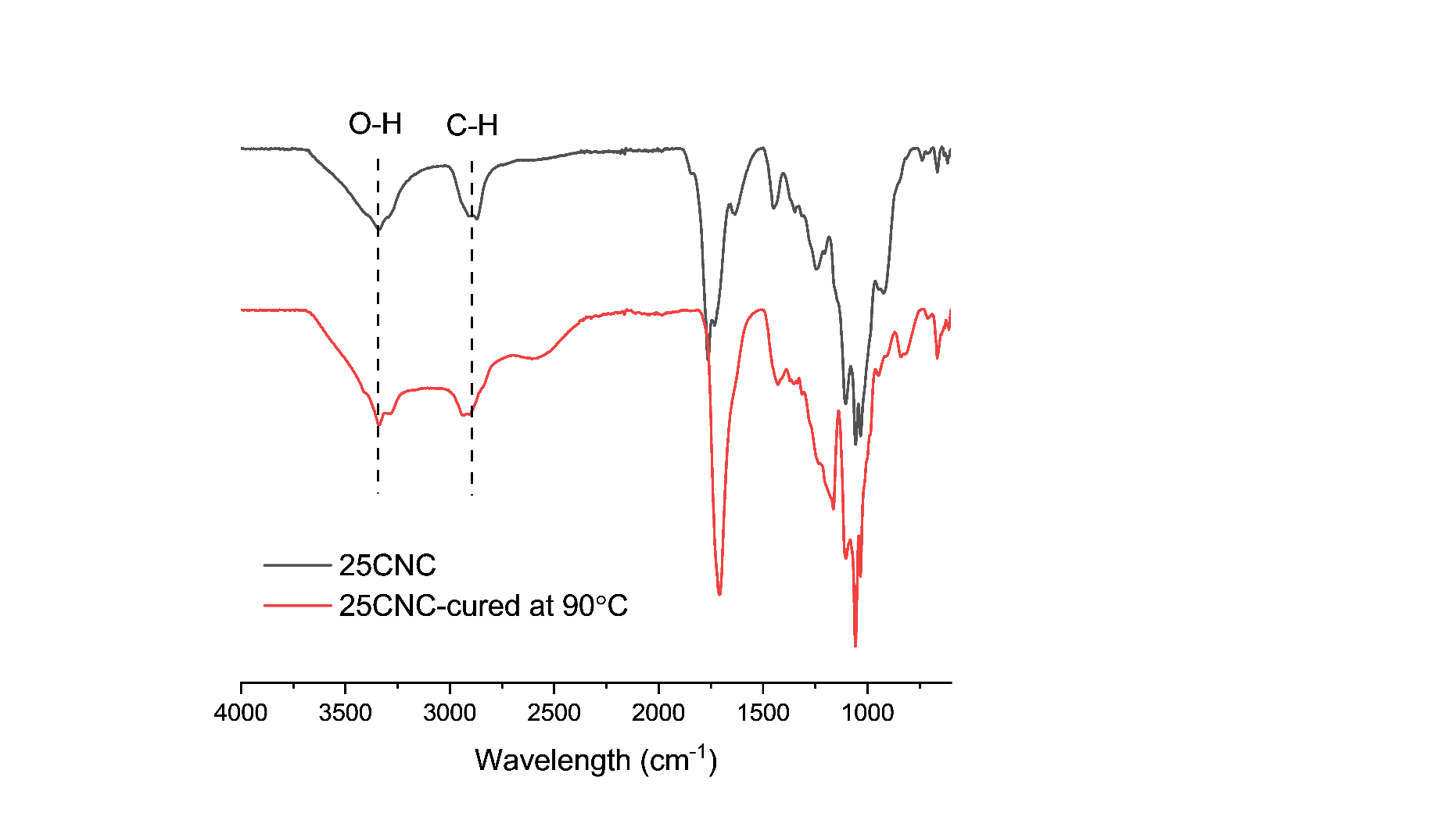


Fig. S1. FT-IR spectra of 250 $℃$ treated and 90 $℃$ treated 25CNC aerogels.


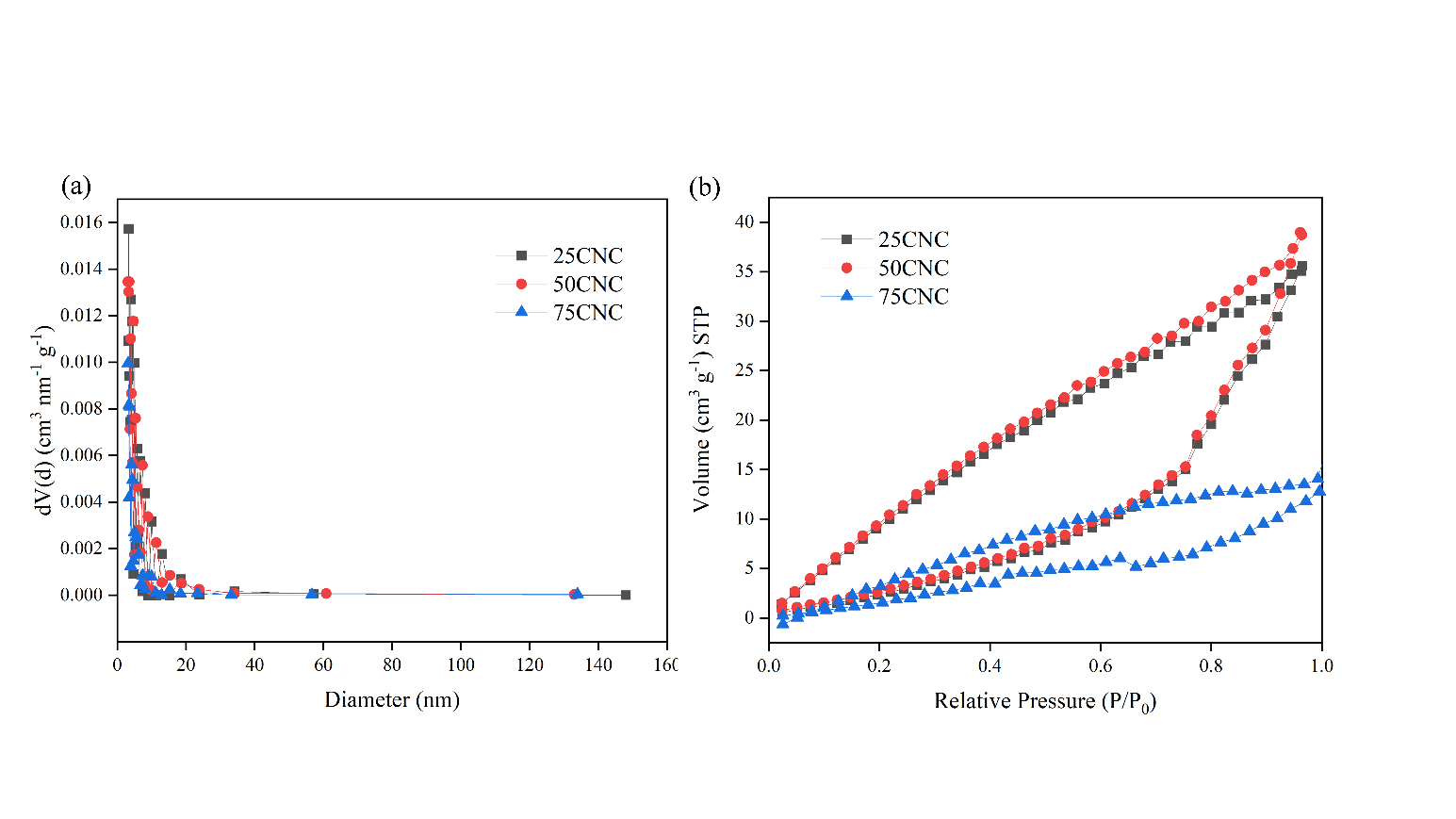


Fig. S2. BET pore size distribution (a) and BET nitrogen adsorption isotherm plot (b).
